# ‘Let it stay in the heart’: cultural and gendered experiences of distress among Syrian refugees in Jordan

**DOI:** 10.3389/fpsyg.2024.1456201

**Published:** 2024-12-18

**Authors:** Jessica E. Lambert, Hala Abutaleb, Rahaf Odeh, Joop de Jong

**Affiliations:** ^1^DIGNITY, International Programmes, Copenhagen, Denmark; ^2^Information and Research Center, King Hussain Foundation, Amman, Jordan; ^3^Amsterdam University Medical Center, Amsterdam, Netherlands

**Keywords:** Syrian refugees, idioms of distress, cultural expressions, thematic analysis, gender

## Abstract

**Objective:**

This study explored cultural and gendered experiences of distress among Syrian refugees in Jordan to inform mental health and psychosocial support services with the population. We sought to understand perceived causes of distress, salient expressions used to describe distress, and ways of coping.

**Methods:**

Eight focus group discussions (FGDs) were conducted with adult Syrian refugees (four male, four female). Gender-matched Jordanian qualitative researchers facilitated the FGDs. Transcripts were analyzed in Arabic using thematic analysis and validated through a final review of translated data.

**Results:**

Four key themes were identified related to participants’ experience of distress, perceived causes and consequences, and gender differences (and similarities) in expression and coping. Results also highlighted the complexity of terminology used, and challenges in rank ordering expressions as most salient.

**Conclusion:**

Results hold implications for adapting mental health and psychosocial interventions with the population to be more gender sensitive and culturally relevant.

## 1 Introduction

Since the start of the Syrian conflict in 2011, more than 1.3 million Syrians have sought refuge in Jordan (Karasapan, 2022), with current estimates of more than 700,000 registered refugees (United Nations High Commissioner for Refugees, 2024). Just under 20% of Syrians in Jordan live in refugee camps, with the majority residing in urban areas such as Amman, Irbid, and Al Mafraq (United Nations High Commissioner for Refugees, 2024). Daily life is challenging for the majority; more than two-thirds are living in poverty (Obi, 2023), with pervasive food insecurity (World Food Programme, 2024). Limited opportunities for employment have forced many families to take drastic measures such as withdrawing children from school, sending family members to beg, and arranging early marriages for girls to cope with financial hardships (United Nations High Commissioner for Refugees, 2024).

Living in these dire conditions, along with a history of exposure to war-related violence and other adversities, has resulted in a significant mental health burden among the population (Hendrickx et al., 2020; Maconick et al., 2020; Sa et al., 2022). Although rates of common mental disorders (CMDs) among Syrian refugees have varied considerably in the literature, research generally suggests high prevalence of anxiety, depression, and posttraumatic stress disorder (PTSD) (Acarturk et al., 2021; Hendrickx et al., 2020). Qualitative studies on the phenomenological life experience of Syrians in Jordan (Bunn et al., 2023; Maconick et al., 2020; Rizkalla et al., 2021) highlight the perceived health impacts of chronic stress (Maconick et al., 2020), the profound sense of loss from displacement (Bunn et al., 2023), and a deep longing to return home (Rizkalla et al., 2021). To effectively address the psychosocial difficulties experienced by the population, it is crucial to incorporate an understanding of the socio-cultural context in which they arise.

The importance of attending to cultural differences in how psychological distress is understood and expressed among displaced populations is widely recognized (Cohen, 2023; Kohrt et al., 2014; Wells et al., 2018). The most recent version of the Diagnostic and Statistical Manual of Mental Disorders, the DSM-5 [American Psychiatric Association (APA), 2013], the term cultural concepts of distress was included for the first time. CCDs are defined as “the ways cultural groups experience, understand, and communicate suffering, behavioral problems, or troubling thoughts and emotions” [American Psychiatric Association (APA), 2013, p.833]. This includes the common expressions or idioms that members of a culture use to communicate various problems or concerns, as well as how individuals interpret their symptoms, understanding the causes, impacts, and potential solutions (Lewis-Fernández and Kirmayer, 2019). Integrating CCDs into mental health and psychosocial support strategies not only honors cultural differences but also has the potential to lessen stigma about mental health interventions and enhance effectiveness (Benish et al., 2011; Hall et al., 2016).

In 2015 UNCHR commissioned a report that provided an overview of terminology and cultural belief systems among Syrians to guide mental health and psychosocial support (MHPSS) work with the population (Hassan et al., 2015). In addition to the identification of common terms and idioms used to describe experiences related to mental wellbeing, these authors described the culturally based concept of the self as being connected to others and to God, and an understanding of wellbeing that is rooted in religious belief. However, most of the published research with Syrian refugees has been centered on medicalized mental health concepts from the Global North, with an emphasis on PTSD and other CMDs. Relatively less attention has been directed toward culturally based understanding of distress and wellbeing (Bjertrup et al., 2018; Wells et al., 2018).

To address this gap, we conducted a qualitative study with Syrian refugees in Jordan to better explored cultural concepts of distress, focusing on perceived causes of distress, common expressions used to describe distress, and common coping strategies. This study was the first step in a larger project aimed at informing the development and cultural adaptation of an MHPSS intervention for helping refugees cope with the impacts of prolonged displacement. We sought to identify the most salient expressions so they could be used in the content of the intervention manual and incorporated into a locally meaningful measure of distress, complementing existing translated measures of CMDs that are commonly used in research of the population.

We used the report by Hassan et al. (2015) as a starting point, reasoning that the extended period of displacement—over a decade for many— combined with exposure to psychological concepts from the Global North through the widespread activities of international NGOs, may have influenced the ways in which Syrian refugees understand and express their psychological symptoms. We were further interested in whether there would be gender differences in how emotions were understood, expressed, and dealt with that might have important implications for engaging males and females in MHPSS.

## 2 Methods

### 2.1 Design and ethical considerations

This study employed a qualitative research design, utilizing eight focus group discussions (FGDs) to explore the expressions of distress among Syrian refugees in Jordan. This design was chosen because it allows for in-depth exploration of complex social and behavioral phenomena through interaction and dialog among participants. Focus groups provide a platform for participants to collectively reflect on their experiences, potentially revealing cultural norms and shared understandings that may not emerge in one-on-one interviews. The FGDs were segregated by gender to ensure comfort and openness, with four groups consisting of male participants and four groups consisting of female participants. Each group was facilitated by two researchers of the same gender as the participants. The FGDs took place in July and August 2023.

Ethical approval was obtained from the Institutional review board at Princess Sumaya University for Technology in Amman, Jordan. Informed consent was obtained from all participants, ensuring they were fully aware of the voluntary nature of their participation, the purpose of the study, and the confidentiality measures. Participants were assured that their involvement in the study would not affect their access to services or benefits. Additionally, a psychologist was available to provide support if participants needed assistance during or after the FGDs.

### 2.2 Research team

The study design was developed by JEL and JdJ as part of a larger project on tailoring mental health interventions to Syrian refugees, funded through the R2HC initiative of the ELRHA Foundation. JEL holds a Ph.D. in Counseling Psychology and over 15 years of experience in conducting research and providing mental health support to populations facing adversity. JdJ Joop de Jong, MD, PhD, is a psychiatrist and psychotherapist. He is Emeritus Professor of Cultural Psychiatry and Global Mental Health at Amsterdam University Medical Schol, with more than three decades of experience with conflict affected populations.

HA, the acting research manager at IRCKHF, holds a master’s degree in comparative literature and has extensive experience in qualitative research and community consultation. HA conducted the focus groups for female participants, alongside female colleagues from the IRCKHF. Additional colleagues supported data collection but were not further involved in the study. HA led the data analysis of Arabic Transcripts. RO, a project assistant at DIGNITY, joined the research team after the interviews were conducted. She reviewed the translated transcripts for accuracy in meaning, and checked the interpretation of results in the manuscript to ensure it was consistent with original data. RO and members of the research team from IRCKHF are Jordanian with experience working with Syrian and other refugee populations in Jordan.

### 2.3 Setting and recruitment

The study was carried out in the Jordanian cities of Amman, Zarqa, Mafraq, and Irbid. These locations were selected to ensure a diverse representation of participants from different urban areas where there are high numbers of refugees. Inclusion criteria required participants to be Syrian refugees aged 18 years or older. Participants were recruited in collaboration with staff from the Institute for Family Health (IFH) centers located in Amman, Zarqa, Mafraq, and Irbid. Health center staff were asked to nominate individuals who were engaged in their communities and who would likely offer valuable insights into common difficulties faced by Syrian refugees, as well as reflect on coping strategies and expressions of distress. Staff were instructed to identify individuals who were not currently experiencing high levels of distress and who felt comfortable discussing sensitive topics related to mental health and psychosocial wellbeing. Efforts were made to recruit individuals from a range of age groups to ensure diversity in perspectives.

Identified individuals were approached face-to-face or via telephone by HA or another researcher from IRCKHF and asked to take part in the study. A majority of those who were invited consented to taking part in the study. Whereas the recruitment process prioritized selecting participants who could meaningfully contribute to the study, participant availability and convenience also played a role. Unfortunately, the exact number of individuals who declined to participate and their reasons for doing so were not systematically recorded, which represents a limitation of the study.

### 2.4 Participants and procedure

The final sample included 81 participants (see Table 1 for an overview of FGDs by gender and location). Participants ranged in age from 19 to 60 years old. Approximately 80% were married, with the remainder being single or widowed. The participants had varying educational backgrounds; 60% of participants had some form of formal education, ranging from primary to university degrees. Younger participants were more likely to have completed higher education. Most were unemployed (70%), with some (20%) having informal jobs and a small percentage (10%) holding full-time jobs. Around 30% of participants reported chronic health conditions, such as diabetes, hypertension, and mental health issues.

**Table 1:** Location and gender for eight focus group discussions (*N* = 81).

Location	Female FGDs	Male FGDs
East Amman	FGD01 (*n* = 10), FGD02 (*n* = 10)	N/A
Zarqa	N/A	FGD03 (*n* = 10), FGD04 (*n* = 10)
Mafraq	FGD05 (*n* = 12)	FGD06 (*n* = 9)
Irbid	FGD07 (*n* = 10)	FGD08 (*n* = 10)

The FGDs consisted of 8–10 participants in each location (see Table 1), each led by two facilitators. Each discussion lasted approximately 2 hours and was conducted in Arabic. The discussions were audio-recorded, and field notes were taken to capture non-verbal cues and group dynamics. A semi-structured interview guide designed for this study was used to explore participants’ experiences of distress and the expressions they use to describe these experiences. The guide aimed to capture a wide range of emotional, cognitive, and physical symptoms associated with distress and how these symptoms affected daily life. The interview guide was pilot-tested with two participants prior to full use to ensure clarity and cultural appropriateness.

At the start of each discussion, participants were informed that their input was part of a larger project aimed at developing a psychosocial intervention for Syrian and other refugee populations experiencing distress. Ground rules were reviewed, and participants were encouraged to share their thoughts freely, with the understanding that there were no right or wrong answers. Facilitators clarified that participants could choose not to respond to any question if they wished.

The interview guide included questions that asked participants to describe the types of distress commonly experienced in their community, including thoughts, emotions, and physical sensations. If participants initially focused on practical problems, such as financial issues or unemployment, facilitators redirected the conversation toward exploring the emotional, cognitive, and physical aspects of distress. Probes were used to gather additional information on symptoms.

Participants were also invited to list common idioms and expressions used in their community to describe distress. For each expression, facilitators asked follow-up questions to understand the meaning, context of use, perceived causes, coping strategies, and whether the expression was used differently by men and women. This process allowed for a detailed exploration of how distress is linguistically expressed and culturally framed.

After generating a list of expressions, the second interviewer cross-checked the expressions discussed with a pre-prepared list from Hassan et al. (2015). For any expressions that were not spontaneously mentioned by participants, facilitators asked whether participants were familiar with these expressions and followed the same process of asking about meaning, context, causes, coping, and gender-specific use.

The final part of the discussion focused on how distress affects people’s daily activities, including self-care, family responsibilities, and community participation. Participants provided specific examples of behaviors that may be impacted by distress, such as changes in eating habits, caregiving, or involvement in community life.

### 2.5 Data analysis

Thematic analysis was conducted following the guidelines of Braun and Clarke (2006), identifying key themes and comparing responses between genders. Two bilingual researchers (Arabic and English) analyzed the transcripts. Separate Excel sheets were created for each interview question, with sections for male and female groups, to organize the data from the eight FGDs. Codes were developed and revised in an iterative process, beginning during data entry and further refined after reviewing all data in its entirety.

Thematic analysis involved reviewing coded data to identify patterns and relationships among keywords, which were then grouped into broader thematic categories. The structured coding tree reflected the relationships between themes, facilitating the comparison of responses between male and female participants.

Themes generated from the analysis were checked against the summary notes taken during each FGD to ensure consistency. As a final step in ensuring the accuracy and fidelity of the analysis, the first author reviewed the translated transcripts and session summaries. For this round of analysis, the data were organized using Atlas.ti. This review served as an audit to confirm the consistency and accuracy of the translation and the alignment of the English summaries with the original Arabic discussions (Table 2).

**Table 2:** Themes, associated expressions, and gender considerations

Theme	Idiom in Arabic	Transliteration	Translation	Associated Emotions	Gender Similarity/ Difference
Fear and Hopelessness in Protracted Displacement	نفسي مخنوقة	Nafsi makhouka	My psyche/soul is suffocating	Emotional exhaustion, overwhelm, fear, hopelessness	Used by both men and women
	تعبان نفسيا	Taeban nafseyan	Tired or fatigued soul	Physical and emotional fatigue	Used by both men and women
Financial Hardship Underpins Psychological Distress	تعبان نفسيا	Taeban nafseyan	Tired or fatigued soul	Physical and emotional fatigue	Used similarly by both genders, but men linked it more specifically to inability to provide financially.
	حاسة الدنيا مسكرةبوجهي	Hases eddenia msakkra bwishi	I feel the world is closing in front of me	Overwhelm, feeling trapped, helplessness; feeling a situation is hopeless	Mostly used by men to describe the feeling of losing control over their situation, but occasionally used by women in contexts of family pressures.
Internalization of Distress and Emotional Restraint	خليها بالقلب تجرحو لا تتطلع لبرا وتفضح	Khaliyha bialqalb tajrah wala tatlue libirana watafdah	Let it stay in the heart and injure rather than exposing me	Emotional restraint, internalization of emotions	Expression used specifically by women, but the theme discussed in all groups
	و الشكوى لغير الله مذلة	Alshakwaa lighayer allah mudhila	Complaining to anyone but God is humiliating	Emotional suppression, resignation	Used by both genders. Among males appeared to be linked with a sense of futility because others cannot help. With women there was a sense of protection of family.
Finding Solace in Religion and Seeking Joy in Everyday Life	حسبي الله ونعم الوكيل	Hassbi Allah wa Na'im al-Wakeel	God is sufficient for me and the best guardian	Faith as a source of strength and comfort	Used by both genders as a source of comfort.
	تغيير الأجواء	Taghyeer al-Ajwaa'	Changing the atmosphere	Shifting mood or environment through music, walking, etc.	Used only by women, who described activities such as playing music, going for a walk, or talking with friends as ways to cope with distress.

## 3 Results

We identified four interrelated themes from the focus group data: (1) Protracted displacement brings fear and hopelessness, (2) Financial hardship underpins distress, (3) Internalization of distress is common, and (4) Religion and connection buffer adversity. Themes address how cultural values shape expressions of vulnerability, strategies for coping with distress, and the pivotal importance of attending to gender. The four themes are elaborated below. Themes and expressions used are shown in Table 2.

### 3.1 Protracted displacement brings fear and hopelessness

The focus group discussions underscored how protracted displacement in Jordan has instilled a pervasive, interlinked sense of fear and hopelessness. Although a multitude of expressions were used to describe the emotional toll of living protracted displacement, the effects of combined fear and hopelessness was expressed by both males and females with the idioms “Nafsi makhouka (my psyche or soul is suffocating)” and “Taeban nafseyan (tired or fatigued soul).”

Participants from both genders voiced concerns about the ongoing crisis in Syria, the unstable nature of humanitarian aid, and their uncertain futures. One female participant from FGD1 captured the essence of this: “We live in fear, fear of the unknown, not sure whether or not we are staying, not sure if our parents in Syria are well, not sure if our kids will have a future here.” A second participant in FGD1 elaborated:

…because I am far from my parents and my mother, I always have this fear… I cannot drive to them or see them, and I cannot visit them, so that’s stress in itself, not to mention the stress of the rest of life and living… fear always accompanies me.

Living in displacement was frequently described as the root cause of fear and other emotional distress. As one female participant explained (FGD2):

The exile made us this way, and we blame it on exile. We’ve experienced stress in our country, but it’s even more when you have no family to turn to when you are tired and there’s no possibility for you to turn around and go back. Themes and expressions used are shown in Table 2.

Male participants noted that the lack of security contributes to an underlying uneasiness that permeates daily life. “There is no sense of security because our future is not known, we have a fear that 1 day the [humanitarian aid] will stop” (FGD6). Concerns about legal status were also prominent, with a participant from FGD3 expressing: “…You are a refugee; at any moment they might deport you.”

Concerns for children’s safety and prospects were shared across genders. Female participants from FGD5 reported bullying in schools affecting their children’s attendance, while a male from FGD3 shared his parental fears:

I left my home, my homeland, and my country so that my son does not get hurt. I am not afraid for myself, but the biggest fear is for my children, if my son suffers, I multiply my suffering by a million.

Another male participant lamented the long-term impact on youth, stating, “our main goal is our children, but you ruined entire generations… my girls… 0.15 and 16 years old, they tell us ‘we have no future’.”

Gender-specific experiences related to life in displacement also emerged, with men but not women reporting stigma and discrimination related to their refugee status. One man from FGD3 recounted feeling demeaned when referred to as a Syrian refugee, “I feel like he’s insulting me, honestly… I feel shameful.” Another from FGD4 described a particularly humiliating incident: “when we were coming out of the mosque, I was told by a man that he would pay 1,000 dinars and send me back to Syria with my whole family.” Such encounters frequently evoked feelings of deep frustration and shame, contributing to the overall sense of hopelessness.

### 3.2 Financial hardship underpins distress

Financial hardship emerged as a related but distinct theme, particularly pronounced in the narratives of male participants who often linked their mental health struggles directly to economic challenges. The expression “fatigued soul” was again raised across groups of participants to indicate the profound exhaustion and hopelessness about the financial situation. For men in particular, the feeling of pressure for needing to provide for their families and being unable to do so was expressed with “Hases eddenia msakkra bwishi (I feel the world is closing in front of my face).” Discussions frequently centered on the severe implications of financial difficulties on personal wellbeing and family dynamics. One male participant from FGD8 encapsulated the sentiment shared by many: “Ninety percent of our problems are rent, bills, and living expenses, meaning there is no income, there is no job, there is no work, there is only unemployment.” This sentiment reflects the pervasive financial anxieties and loss of status affecting men, who have a culturally proscribed role as providers for the family.

The inability of men to fulfill their role of provider due to high living costs and scarce employment opportunities seemed do drive their despair. A male participant from FGD6 articulated the psychological impact of financial pressures, stating, “When the head of the family is unable to provide for his family, he feels vulnerable.” Further compounding these challenges, a participant from FGD3 shared a difficult conversation with his daughter, underscoring how family pressures the father to provide: “You do not bring in money, how do you want me to study? Educate me and create opportunities. I have no future. When you want me to marry, will you sell me?” This exchange also underscores the broader implications of financial hardship on the family’s outlook on life and opportunities.

Financial problems were mentioned as key drivers of marital problems. One participant captured the stress on marital relationships and the additional pressure on men to meet traditional expectations within the household:

A man is usually required to provide… Here, if a woman is nurturing and kind and knows how a man suffers, she will stand by him and be patient, but if she has no patience, she will make his life at home more hell than out of the home.

The lack of opportunities has contributed to the loss of purpose and ambition coupled with a loss of vision for the future. A participant from FGD4 lamented:

There is no ambition, there is no creativity, there is nothing. You do not wake up in the morning and say, ‘I want to benefit society with something’… even a young man does not think about getting married, he does not think about opening a house, he does not think about his life project, work has been cut off… there is no hope for him.”

Some participants attributed distress related to decline in socioeconomic status in displacement. A male in FGD 3 for example noted he could never earn as much or go as far with his career as he did in Syria. This sentiment was echoed by another participant in the group who stated: “My father was a colonel in the army and a high-ranking officer, and we had money, but here my situation is trash.” Some men reported that such financial pressures had led to extreme outcomes, including divorce and suicidal thoughts, highlighting the profound personal and social ramifications of economic struggles and loss of sense of self.

Financial concerns also surfaced in women’s discussions however, they did not dominate the conversations as they did for men. One participant expressed helplessness noting: “The dire financial situation makes you feel trapped, meaning you do not know how to get out of the distress you are in (FGD5).” Only two participants from FGD2 explicitly identified money as their central concern, with one saying, “The thing that bothers me the most is when there’s no money. When there’s money, everything is fine.” Another participant added, “If you do not have money, you cannot give or pay for anything, and you just have to live.” A third participant subtly contradicted this focus on financial issues, suggesting, “There is more than just money,” explaining that their experiences of distress were influenced by a variety of factors beyond financial hardship alone.

### 3.3 Internalization of distress is common

Captured by expression “Let it stay in the heart and injure rather than going out and exposing me” khaliyha bialqalb tajrah wala tatlue libirana watafdah (Female FDG7), this theme reflects a cultural or personal preference to bear emotional pain silently rather than expressing it externally, showing vulnerability, and facing potential societal judgment. The theme emerged in both male and female FDGs, but there were clear gender differences. Women described themselves as keeping emotions inside, particularly in their roles as emotional anchors of the family. However, there was a general sense that women are more open about expressing vulnerable feelings than men, in certain situations, like when confiding in a trusted friend. Women across groups spoke at length about the societal expectations placed on men, which often discourage open expressions of vulnerability. For men, this theme was reflected in their descriptions of masculinity and was indirectly highlighted by the group dynamics, where discussions about emotions were consistently redirected toward practical issues or expressions of anger. Interestingly, in the discussions with both males and females in the groups, as detailed below, the potentially detrimental consequences of internalized emotions were mentioned.

Although both female and male participants noted that women are more open about their emotional states, there remains a tendency for women to internalize distress. Some women shared a personal preference for keeping emotions to themselves, noting “it’s better to shut down than to open up” (FGD7) and “complaining to someone else is humiliating” (FGD2). A tendency to internalize also came through for women in descriptions of coping strategies like going to sleep or isolation from others. A subtheme that emerged for women was the tendency to internalize feelings when it pertains to family wellbeing. Women might discuss their emotional states more freely in certain contexts but still choose to ‘keep it in the heart’ to protect the family. However, this pressure on women to hold the emotions of the household together has consequences for their wellbeing. One participant explained:

A mother, as much as she can, keeps her struggles and the burdens of her husband, children, and home to herself. She endures as much as she can. For example, my mom carries a lot, and then she eventually breaks down. We all become scattered because she has given us so much. This leads to a sense of desperation and worry because the mother is the foundation of the home, so if the foundation is damaged, it’s a disaster (FGD7).

A second women in FGD7 elaborated on how this dynamic can lead to unhealthy coping “… she tolerates and tolerates and tolerates and then she overindulges [in food and sweets].”

Despite the descriptions of holding in emotions with and about the immediate family, women generally noted that they were more likely than men to share emotions and the rich vocabulary of emotional expression was evident in each of the discussions. As explained by one woman, “opening up to someone, that’s the best thing to do, to not be secretive and keep something in your heart.” Other female participants described the detrimental impacts of internalizing feelings: “silence is a killer, you have to release the energy you have, whether you are happy or angry, you have to express it.” Others expressed the belief that suppression of emotions can lead to physical ailments such as high blood pressure, stroke or diabetes, and mental health struggles such as depression.

Female participants also spoke about men and their emotional expression, shedding light on the societal expectations that shape gender differences. They highlighted the pressure on men to conform to ideals of masculinity, which often discourage them from openly expressing their feelings and vulnerability. One woman explained “A father is the source of strength for his family, how can he allow his family to see him exhausted? In general, males are raised not to show fear, fatigue, or weakness. Society raised them to be that way (Female participant FGD7).”

This societal expectation of suppressing emotions can lead to counterproductive externalizing outlets for men such as yelling, breaking things, or in extreme cases becoming physically violent. When describing the pressure on men to be providers and problem solvers, with no outlet for expressing emotions, one participant noted:

They face so much pressure, most pressure is on males, females as well but males it’s a lot. Men are tired from everything, physically and psychologically. They are tense as well. They sometimes hit because they do not know what to do (Female FGD02).

Male participants reported a withdrawing from family interactions and seeking solitude when distressed, typically refraining from sharing their emotions with others. Some described this as a protective instinct, preferring to shield their families from the stresses associated with their refugee status and financial responsibilities. This protective stance often results in them distancing themselves or shutting down emotionally, which some seemed to indicate was the best option they could choose. When discussing their feelings, men commonly described physical symptoms of stress such as headaches, muscle tension, fatigue, and digestive issues, indicating a tendency to focus on physical rather than emotional expressions of distress.

The preference for avoiding discussion of emotion was evidenced in the process of the FGDs with men where participants tended to tell long stories about specific events to illustrate a point, often unrelated to the question asked by facilitators, focusing mainly on the context rather than their inner experience. Some male participants also questioned the function of the FGDs, expressing fatigue with social interventions that brought little relief for Syrian refugees in Jordan, noting that humanitarian efforts should instead focus on improving their financial situation. This sentiment was illustrated by a participant from FGD5 who asked, “Why do you bring up topics that make our hearts ache? I mean, not all of our stories can be shared.” A participant from FGD6 stated “Look at our society, it is difficult to find the person you want to complain to because we are in the same situation as each other,” explaining that sharing distress to others who face the same problems is not useful. This sentiment was expressed with the idiom “complaining to anyone but God is humiliating.”

### 3.4 Religion and connection buffer adversity

Despite the overwhelming uncertainty and stress experienced by participants, many have developed ways to navigate daily challenges, finding solace in God and religious practices, connecting with loved ones, and utilizing strategies to mitigate the impacts of adversity. Men predominately identified religion as bringing relief, noting throughout the groups Similarly, male participants used the expression “Hassbi Allah wa na′im al-wakeel” (God is sufficient for me and the best guardian) as a reminder of their faith in a higher power. Women, in contrast, described a wide array of additional coping mechanisms that helped them in to “taghyir aljawi (change the atmosphere)” in daily life.

Participants across groups noted that prayer and reading the Qur’an offered a source of comfort during challenging times. Expressions of thanks to God were reiterated across all FGDs. By seeking refuge in faith, some found a sense of reassurance. One female participant stated: “I feel afraid, I pray, seek forgiveness, and try to calm down… I find solace in remembering to thank God for everything and find tranquility in that” (FGD2).

Women also describe the importance of relationships and meaning making as a way of coping, one participant noting “The love of life, the love of endeavor, and the love of family can give you the energy to move forward.” (FGD1). Underscoring the importance of family, one participant shared that despite ongoing stress she focuses on her family making efforts to live “normally with my children, laughing and playing with them, listening to music” (FGD2). Some expressed the importance of seeking companionship from family members or friends, whether they discuss problems or not.

A common expression in relation to coping mentioned by female participants was the importance of finding ways to “change the atmosphere,” meaning shifting their own internal state or the dynamics in the household. Women approached this in myriad ways, often evoking a playful approach including playing music and dancing in the house, finding ways to laugh, or even putting on a perfume to shift their mood. They also changed the atmosphere by going for a walk or speaking to a friend.

## 4 Discussion

In this qualitative study, we explored cultural concepts of distress among adult Syrian refugees in Jordan, focusing on identifying salient, locally relevant expressions, perceived causes of distress, and ways of coping with distress at for males and females. Four interrelated themes emerged from the focus group data, highlighting the contextual challenges of life in Jordan, the impacts of these challenges, and ways in which people in their community typically cope. There were similarities among the description of challenges related to being a refugee between men and women. Gender differences emerged in relation to emotional expression and coping preferences. The diversity of expressions used to describe emotional experience, made it challenging to rank order expressions by salience. Nevertheless, we identified common expressions used that can be useful for the cultural adaptation of mental health and psychosocial interventions and assessment tools. The four themes are explored in the context of the literature below, followed by potential implications and limitations.

The first two themes that emerged from the focus groups “protracted displacement is characterized by fear and hopelessness” and “financial hardship underpins distress-especially for men” reflect the profound challenges of life in protracted displacement and highlight similarities and differences across genders. Related to the first theme, both male and female participants described life in Jordan as being characterized by pervasive fear and hopelessness. Participants expressed deep concerns about the ongoing crisis in Syria, the unpredictability of resources, and their uncertain futures. These fears were compounded by the separation from family members and the inability to provide security for their children. Consistent with other studies (Bjertrup et al., 2018; Bunn et al., 2023; Sagbakken et al., 2020) male and female participants described how the loss of their homeland, social networks, and the futures they had once imagined for themselves, and their families contributed to this sense of uneasiness and loss of hope.

The second, closely related, theme involved participants’ view that financial hardship is a critical driver of distress, particularly affecting male participants who linked their mental health struggles directly to economic challenges. For men, this theme went beyond just the lack of money, to include the loss of identity and purpose that came from being the economic provider for their families. Both male and female participants described the pressure experienced by men related to the loss of this role and the limited opportunities to rebuild in the context of displacement, which others have linked to a loss of dignity (Wells et al., 2018).

These two interrelated themes can be understood within the framework of social suffering (Kleinman et al., 1997), encompassing the interplay between individual and collective experiences among populations living in adversity. From this perspective, individual suffering is seen in context and is closely linked to broader political, social, and cultural circumstances. Rather than focusing solely on individual psychopathology or distress, social suffering considers the lived experiences of individuals within their societal framework (Kleinman et al., 1997). This approach emphasizes the need to look beyond medicalized or individual perspectives and to incorporate the life narratives of those affected by distress, recognizing that their suffering often stems from larger socio-political issues (Maconick et al., 2020).

Themes consistent with social suffering have emerged in other qualitative studies of Syrian refugees in Jordan (Maconick et al., 2020; Rizkalla et al., 2021; Wells et al., 2018). For example, in a study of Syrian refugees with chronic health conditions in Jordan, Maconick et al. (2020) found participants closely linked their emotional experience with the context, viewing individual distress as inseparable from the collective experience of war and displacement. Similarly, in the ecological model of adaptation developed by Wells et al. (2018) from a qualitative study of Syrian refugees in Jordan, the broad experience of past adversity coupled with ongoing stress was central to understanding how Syrian refugees were adapting to life in Jordan.

Participants used several different expressions to describe the inner experience associated with social suffering described above. In addition to the direct translations of words commonly used in mental health in the Global North, like depression, anxiety, and fear, participants used the expressions “Nafsi makhouka (my psyche or soul is suffocating),” “Taeban nafseyan (I have a tired or fatigued soul)” and “Hases eddenia msakkra bwishi (I feel the world is closing in front of my face).” Participants noted that other expressions outlined by Hassan et al. (2015) were also used by people in their communities, noting that the choice of terminology is related to individual personality, circumstances that might evoke different emotions, as well as differences in education, and region of origin. Some participants expressed difficulty pinpointing exactly when or why different phrases would be chosen, noting it is a personal preference how one expresses themselves.

In our exploration of terminology used to express inner emotional experience, a third theme related to the internalization of distress and restraint in sharing emotion, encapsulated by the expression: “Let it stay in the heart and injure rather than going out and exposing me (khaliyha bialqalb tajrah wala tatlue libirana watafdah).” Although women were more likely than men to share their feelings in trusted contexts, they described their roles as emotional anchors of the family and keeping emotions inside to protect their families. Women described enduring some emotional pain silently, maintaining a facade of strength to shield their family members from additional stress. This finding resonates with Lokot (2021), who found a tendency for Syrian women to internalize problems to preserve family honor, using idioms like “keep the floor covered” and “complaining to anyone but God is a disgrace.” However, it is also important to note that several women in our study also expressed how internalization of emotion was often linked to unhealthy coping mechanisms and physical health issues, with participants expressing the belief that suppressed emotions could lead to ailments such as high blood pressure, diabetes and stroke. These participants expressed the importance of sharing joy and sorrow with close friends as a way of releasing stress. Thus, when noting cultural trends, it is also important to recognize individual variability.

In relation to emotional expression among men, both men and women emphasized the societal expectations of masculinity, discouraging open expressions of vulnerability. This dynamic often resulted in men withdrawing from family interactions, or in extreme cases engaging in violent behavior. Men noted that internalization could result in anger, reflecting the pressures of dealing with the stress of displacement and tensions around being unable to fulfill their role. Similarly, Lokot (2021) noted themes related to a desire to keep problems private, that asking for help may be seen as shameful, and men felt a sense of needing to shield their families from the emotional strain they experienced because of financial problems. Wells et al. (2018) also noted that self-reliance is central to understanding masculinity among Syrian men. Thus, while “keeping it in the heart” among women may be more closely related to the protection of the family, among men it seems closely tied to a cultural expectation of stoicism.

The fourth and final theme encompassed the way people cope with social suffering associated with life in displacement. Despite the overwhelming uncertainty and stress experienced by participants, many have developed ways to navigate daily challenges, finding solace in God and religious practices, connecting with loved ones, and utilizing strategies to mitigate the impacts of adversity. Men predominantly identified religion as a source of relief, with many noting that prayer and reading the Qur’an offered comfort. Expressions of thanks to God were reiterated across all focus group discussions. Like findings from Alzoubi et al. (2019), men in our study also engaged in avoidance as a coping style, primarily leaving the house when upset to avoid conflict.

In addition to finding solace in faith, women described various coping mechanisms, including maintaining relationships and finding meaning in daily life. For example, one female participant emphasized the love of life, endeavor, and family as sources of energy. Women often mentioned efforts to live normally with their children, engaging in playful activities like laughing and playing music to create a positive atmosphere. Consistent with findings in other research (Alzoubi et al., 2019), female participants in our study also sought companionship from family and friends as a means of coping. Additionally, they used various strategies to change their internal state or household dynamics, such as going for walks, speaking to friends, and finding joy in small moments to shift their moods.

### 4.1 Implications

Our findings hold implications for the types of interventions that might be relevant in this context, as well as considerations for adapting interventions to the cultural context. It was evident in our study that socioeconomic deprivation and loss of traditional roles are contributing to mental health difficulties. For this reason, interventions that address socioeconomic and mental health dimensions of refugees’ experiences are needed. The descriptions of social suffering provided by our participants point to the relevance of MHPSS interventions that support people’s agency (Wells et al., 2018) and support building community networks (Bunn et al., 2023).

Findings also highlight sensitivity around the expression of distress and discussion of problems and how this might differ for men and women. This has important implications for engaging people in mental health services. Men expressed reluctance over the value of sharing difficulties, particularly among others who shared similar problems, because it would not likely lead to a solution. It could be that interventions aimed at resolving practical problems, like Problem Management Plus (PM+; WHO), are well suited to men in this context. One trial of PM+ in Jordan showed promising results, but the researchers had difficulties engaging men, who comprised only 25% of the study’s sample (Bryant et al., 2022). Women, although more open to sharing emotions in certain contexts than men, also expressed a tendency to internalize distress, which could influence engagement in mental health services, particularly in a group setting. It is interesting to note, however, that a core theme from a qualitative study of Syrians in Jordan who had been through a trauma-focused group mental health intervention was that “sharing stories eases pain” (Bunn et al., 2022). This finding suggests that people benefit from sharing experiences in a trusted environment despite a culturally based preference for internalizing emotions.

Our findings also point to the importance of understanding cultural concepts of distress, and recognizing idioms and the meaning they hold for people. It is possible that the idioms of distress have more salience for the refugees than words like depression or anxiety. These expressions may be useful for including in psychoeducation or engaging the community in MHPSS interventions. They may also help to destigmatize mental health and psychosocial interventions and lower the threshold for seeking help.

### 4.2 Limitations and future research

There are several limitations that are important to note. We did not have Syrian refugees on our research team, which is a limitation. Our team was composed of researchers from the Global North and Jordanians, which could have introduced bias in the interview process and interpretation of results. Participants in the FGDs did not identify themselves before each speaking turn, therefore it was not possible to evaluate the degree to which each person contributed to the discussion. Given the study’s qualitative nature, we could not determine the degree to which gender, age, and education level might influence the expressions used. The small sample size typical of qualitative studies may not be representative of the broader population, limiting the generalizability of the results. Additionally, reliance on self-reported data means that findings are subject to the accuracy and honesty of participants’ disclosures, which can be influenced by recall bias or the desire to present oneself in a favorable light.

We recommend future research aims to address some of the limitations identified in this study. In-depth interviews would allow researchers to map associations between specific expressions, emotions, and coping strategies, offering a more granular understanding of how these idioms function in everyday life and how they might be leveraged in mental health treatment. While our study provides valuable insight into the ways in which these expressions are used, the qualitative nature of our data did not allow for a systematic examination of the impact of demographic variables on these linguistic patterns. Future studies could incorporate larger, more diverse samples to better understand how these variables shape emotional expression and coping strategies. Additionally, including Syrian refugees as part of the research team could help mitigate potential biases and ensure a more culturally nuanced interpretation of findings. By deepening our understanding of the cultural and social factors influencing emotional expression, future research could contribute to more effective, culturally adapted mental health interventions. Lastly, it may be beneficial to explore the extent to which there are similarities in expressions among other populations in the region that share a similar cultural background.

## 5 Conclusion

Consistent with other qualitative studies of Syrian refugees in Jordan (Bunn et al., 2022, 2023; Maconick et al., 2020), our findings highlight the profound social suffering associated with life in protracted displacement. Coupled with quantitative research documenting the high prevalence of CMDs, these findings underscore the continued need for MHPSS services. Our results emphasize the importance of culturally adapting interventions to be more acceptable to the population, including sensitivity around the disclosure of emotions and experiences. The lack of resources and uncertainty about the future appear to cause or contribute to pervasive distress. For this reason, in addition to the provision of MHPSS, it is essential to address the social conditions that are contributing to mental health issues.

## Data Availability

The raw data supporting the conclusions of this article will be made available by the authors, without undue reservation.
